# 蛋白质组学与转化医学：用以癌症诊断、预后和疗效预测的分子生物标记物

**DOI:** 10.3779/j.issn.1009-3419.2011.08.12

**Published:** 2011-08-20

**Authors:** William CS CHO, 娟 南

**Affiliations:** 1 Department of Clinical Oncology, Queen Elizabeth Hospital, Hong Kong; 2 天津医科大学总医院，天津市肺癌研究所，天津市肺癌转移与肿瘤微环境重点实验室; 3 香港特别行政区 伊利沙伯医院 临床肿瘤科

**Keywords:** 癌症, 分子生物标记物, 肿瘤蛋白质组学, 蛋白质组学, 转化医学

**“生物医学研究的最新技术进展使鉴定多种分子生物标记物变得更加容易，有助于促进癌症筛查和检测……通过允许医生为患者制定个体化治疗来提高癌症治疗的有效性和安全性。”**

癌症是当今世界的主要致死原因。癌症生物标记物对于良好的治疗效果和远期疗效至关重要，并可降低病痛和疾病相关的社会支出。全世界恶性肿瘤的高负担突显了用以癌症诊断、预后和疗效预测的生物标记物的未尽潜能。迫切需要新型策略以发现癌症生物标记物并将分子诊断从实验研究转化至临床实践^[1, 2]^。

生物医学研究的最新技术进展使鉴定多种分子生物标记物变得更加容易，有助于促进癌症筛查和检测，推进药物研发进程，通过允许医生为患者制定个体化治疗来提高癌症治疗的有效性和安全性^[3]^。蛋白质组学是用以鉴定癌症生物标记物和新型治疗靶标的具前景的技术。肿瘤蛋白质组学的飞跃发展为肿瘤相关生物标记物的发现开辟了新的途径^[4]^。

## 诊断或早期检测的生物标记物

癌症的早期检测是提高恶性肿瘤总生存期的途径之一。MALDI-TOF/TOF检测发现，与无瘤对照组相比，体积较小（≤2 cm）的肝癌（hepatocellular carcinoma, HCC）患者的血清波形蛋白明显过表达。进一步研究证实波形蛋白单独或与甲胎蛋白联合均可作为检测体积较小的HCCs的潜在替代标记物^[5]^。对HCC组织进行基于二维凝胶电泳的比较蛋白质组学分析发现，苯酚磺基转移酶（sulfotransferase, SULT1A1）的下调与晚期国际抗癌联盟分期和高血清甲胎蛋白水平密切相关。SULT1A1可能是筛查早期HCC的有效生物标记物，并有助于预测HCC患者的临床疗效^[6]^。

一项采用标准免疫蛋白质组学技术和Luminex为基础的直接捕捉免疫念珠试验的基于多种肿瘤相关自身抗体（包括膜联蛋白Ⅰ、膜联蛋白Ⅱ、热休克蛋白70-9B、肌苷-5-单磷酸脱氢酶、磷酸甘油酸变位酶和ubiquillin）的血液检测可从高危人群中筛查出早期非小细胞肺癌（non-small cell lung cancer, NSCLC）。这6个生物标记物联合的误分类率仅为7% ^[7]^。此外，对不同分期的膀胱癌患者尿样的同位素标记相对和绝对定量（isobaric tag for relative and absolute quantitation, iTRAQ）检测表明，载脂蛋白A-Ⅰ（apolipoprotein A-Ⅰ, APOA1）是膀胱癌早期筛查的潜在生物标记物。敏感性和特异性分别为84%和94%^[8]^。

最近，美国食品药物管理局（Food and Drug Administration, FDA）批准了一种卵巢肿瘤的分流方法OVA1^TM^，标志着蛋白标记物从实验室走向临床进程中的里程碑。当与临床评估（如影像和体格检查）联合时，这一基于免疫测定的分析方法（包括2-微球蛋白、APOA1、CA125、转铁蛋白和转甲蛋白）在恶性肿瘤高危女性人群中具有阳性预测价值。OVA1评分可帮助医生决定恶性肿瘤高危患者是否可从转诊至妇科肿瘤治疗中获益^[9]^。

## 预后或转移的生物标记物

预后或转移的生物标记物通过鉴别具有不同风险的个体，有助于癌症患者的分组治疗。最近鉴定出了许多可提供重要预后信息的生物标记物。在一项卵巢浆液性交界性肿瘤和卵巢浆液性癌的全蛋白质组的比较研究中，过氧化物氧还酶1（peroxiredoxin 1, PRDX1）的过表达与浆液性癌的总生存期较短明显相关。另一项多变量Cox分析中，PRDX1阳性癌细胞数＞50%的浆液性癌患者的相对死亡风险为8.74。这些结果提示，PRDX1是卵巢浆液性癌的有效预后生物标记物^[10]^。对比良性膀胱尿路上皮组织和移行细胞癌，膀胱癌相关蛋白的高表达预示着患者预后不良，提示此蛋白染色模式的分类可能具有预后价值。而且，与单一标记物相比，膀胱癌相关蛋白和脂肪细胞型脂肪酸结合蛋白（adipocyte fatty-acid binding protein, A-FABP）的联合与疾病分级和/或分期的相关度更为密切^[11]^。

**“预后或转移的生物标记物通过鉴别具有不同风险的个体，有助于癌症患者的分组治疗。”**

采用激光显微切割、精确质量数和保留时间标签蛋白质组学，有研究报道肝内胆管细胞癌（intrahepatic cholangiocarcinoma, ICC）中的不同蛋白位于各种潜在的肿瘤发生的通路上。通过组织微阵列分析发现，70%的ICC患者中波形蛋白表达增多，而对照组中未见其表达。这些结果提示，波形蛋白在ICC的侵袭中发挥作用，而且是其不良预后的基础^[12]^。有研究对脑膜瘤组织实施SELDI-TOF质谱技术（mass spectrometry, MS）分析以期发现组织侵袭/浸润的新型标记物。磷酸化波形蛋白的增加可能成为鉴别浸润性脑膜瘤和非浸润性脑膜瘤的标记物。敏感性和特异性分别为86.7%和100%^[13]^。

对于NSCLC，通过SELDI-TOF MS检测血清淀粉样蛋白A（serum amyloid A, SAA）发现，与生存期≥5年的患者相比，生存期＜5年的患者的SAA明显增高。SAA水平的增高有可能成为预测NSCLC预后的非侵袭性生物标记物^[14]^。SELDI-TOF MS检测还发现，载脂蛋白A-Ⅱ和SAA是判定转移性肾脏细胞癌患者生存期的独立因素。这两种蛋白联合乳酸脱氢酶（lactate dehydrogenase, LDH）、体力状况和转移位点数目形成了新型预后生存模型。与目前使用的纪念斯隆-凯特灵癌症研究中心（Memorial SloanKettering Cancer Centre）风险模型相比，基于蛋白的模型可能提高对总生存期的预测^[15]^。

细胞培养中的氨基酸稳定同位素标记与液相色谱（liquid chromatography, LC）-MS/MS分析发现，TIMM17A（mitochondrial import inner membrane translocase subunit Tim17-A）是乳腺癌的预后因子。TIMM17A的表达水平与肿瘤进展和生存期直接相关。过表达和siRNA敲除试验证实了TIMM17A在乳腺癌中的致癌活性^[16]^。一种基于LC-MS/MS的无标记定量蛋白质组学方法用以比较原发性结直肠癌（colorectal cancer, CRC）及其淋巴结转移细胞的差异分泌蛋白质组。免疫组织化学的分析结果显示，CRC中的生长分化因子15或三叶因子3的过表达与淋巴结转移相关。它们有可能成为预测CRC转移的生物标记物^[17]^。

鼻咽癌（nasopharyngeal carcinoma, NPC）细胞分泌蛋白质组和组织转录组分析显示，治疗前的血清cystatin A水平越高，则NPC患者的淋巴结分期越高且预后越差。Cystatin A可调节离体NPC细胞的迁移和侵袭^[18]^。有研究发现，NPC患者的真核翻译起始因子4G1（eukaryotic translation initiation factor 4 γ1, EIF4G1）的表达越高，则总生存期越短。采用shRNA敲除EIF4G1的表达，不仅可明显抑制细胞周期进程、增殖、迁移、侵袭和集落形成，而且可显著抑制在体异种移植瘤的生长^[19]^。

## 生物标记物对治疗效益的预测

肿瘤生物标记物的另一重要方面是它们为选择更具靶向性的治疗方法提供了预测价值。蛋白质组学分析显示，14-3-3σ和maspin的下调，以及GRP78和Mn-SOD的上调，均与NPC的放疗耐受显著相关。在识别放疗耐受的NPC患者的放疗敏感度中，四种蛋白联合应用的敏感性和特异性分别为90%和80%。而且，放疗耐受细胞通过过表达14-3-3σ可部分逆转对电离辐射的耐受^[20]^。治疗前活检标本的蛋白质组学和免疫组织化学分析显示，对新辅助放疗或紫杉醇治疗呈现病理完全缓解的乳腺癌患者的免疫信号分子α-defensin呈过表达。对术前接受基于紫杉醇治疗的肿瘤患者的大样本分析显示，手术时的α-defensins与治疗效果相关^[21]^。

反相蛋白阵列检测发现，EGF受体（EGF receptor, EGFR）和两种TGF-β通路蛋白（c-jun-NH_2_-激酶和Smad3）与晚期浆液性卵巢癌患者化疗后CA125的正常化相关。在晚期浆液性卵巢癌中，TGF-β通路信号可能作为化疗耐受的标记物而发挥重要作用^[22]^。

**“肿瘤蛋白质组学揭示肿瘤发生的复杂分子事件以及控制临床上重要的肿瘤行为，如侵袭、转移和对治疗耐受，提供巨大前景。”**

一项Ⅰ期剂量递增研究在转移性CRC患者血浆和组织样本中评估了西妥昔单抗疗效的生物标记物^[23]^。药物蛋白质组学和药物基因组学分析显示，仅*KRAS*野生型肿瘤患者有效，而且与*KRAS*突变型肿瘤相比，*KRAS*野生型患者的无进展生存期更长。这些结果证实，*KRAS*野生型的转移CRC患者更易从西妥昔单抗治疗中获益^[23]^。在采用西妥昔单抗或EGFR酪氨酸激酶抑制剂治疗的CRC患者中，肿瘤EGFR配体RNA水平亦与VeriStrat特征[Biodesix（布鲁姆菲尔德，科罗拉多州，美国）提供的基于8种不同的m/z特征的血清MALDI分类检测]预测的生存期显著相关，联合*KRAS*突变状态可提高对生存期的预测。生物标记物的联合为鉴别更易获益于EGFR抑制剂治疗的不同肿瘤类型的患者提供了临床实践方法^[24]^。采用VeriStrat^®^分析厄洛替尼一线治疗晚期肺癌患者的Ⅱ期研究的生物标本发现，VeriStrat状态和*EGFR*突变与生存期显著相关，而*KRAS*突变与生存期无关。VeriStrat被证实是野生型*EGFR*且不伴有*KRAS*突变的患者采用厄洛替尼一线治疗后生存期的有效预测因子^[25]^。

## 挑战与展望

尽管近几十年来分子生物标记物的发现急剧增加，但将这些生物标记物转化为更有效的患者治疗和更良好的疗效仍存挑战。研发临床上有效的癌症生物标记物还有许多障碍，包括与分析前参数及潜在癌症生物标记物验证相关的技术难题，以及将这些癌症生物标记物转化为临床实践的研发、评估与联合筛查或诊断测试相关的挑战^[3]^。

新兴的组学技术正广泛地用于癌症研究和生物标记物的探索^[26]^。高通量蛋白质组学技术的近期进展以前所未有的速度为人类肿瘤的分子分析提供了新的机遇。肿瘤蛋白质组学揭示肿瘤发生的复杂分子事件以及控制临床上重要的肿瘤行为，如侵袭、转移和对治疗耐受，提供巨大前景^[4]^。随着新的和改进的蛋白质芯片技术的出现，让研发可靠并准确地预测癌症治疗效果的生物标记物变得可行^[27]^。可以预见，未来蛋白生物标记物应用可能需要微型化和自动技术。多种生物标记物的联合可使检测更为精确。已有生物标记物联合新型分子标记物将会是研发癌症管理的治疗诊断方法的新兴趋势。分子癌症生物标记物在转化医学中不断向前发展，但距离成功还有漫漫长路。

## Financial & competing interests disclosure

The author has no relevant affiliations or financial involvement with any organization or entity with a financial interest in or financial conflict with the subject matter or materials discussed in the manuscript. This includes employment, consultancies, honoraria, stock ownership or options, expert testimony, grants or patents received or pending, or royalties.

No writing assistance was utilized in the production of this manuscript.

## References

[1] Cho WC (2010). Conquering cancer through discovery research. IUBMB Life.

[2] Cho WC (2011). Molecular diagnostics for monitoring and predicting therapeutic effect in cancer. Expert Rev. Mol. Diagn..

[b3] 3Cho WC. Cancer biomarkers (an overview). In: *Methods of Cancer Diagnosis*, *Therapy and Prognosis (Volume 7)*. Hayat MA (Ed. ). Springer, Berlin, Germany, 21-40 (2010).

[4] Cho WC, Cheng CH (2007). Oncoproteomics: current trends and future perspectives. Expert Rev. Proteomics.

[5] Sun S, Poon RT, Lee NP (2010). Proteomics of hepatocellular carcinoma: serum vimentin as a surrogate marker for small tumors (< or =2 cm). J. Proteome Res..

[6] Yeo M, Na YM, Kim DK (2010). The loss of phenol sulfotransferase 1 in hepatocellular carcinogenesis. Proteomics.

[7] Farlow EC, Patel K, Basu S (2010). Development of a multiplexed tumorassociated autoantibody-based blood test for the detection of non-small cell lung cancer. Clin. Cancer Res..

[8] Chen YT, Chen CL, Chen HW (2010). Discovery of novel bladder cancer biomarkers by comparative urine proteomics using iTRAQ technology. J. Proteome Res..

[9] Fung ET (2010). A recipe for proteomics diagnostic test development: the OVA1 test, from biomarker discovery to FDA clearance. Clin. Chem..

[10] Chung KH, Lee DH, Kim Y (2010). Proteomic identification of overexpressed PRDX 1 and its clinical implications in ovarian carcinoma. J. Proteome Res..

[11] Moreira JM, Ohlsson G, Gromov P (2010). Bladder cancer-associated protein, a potential prognostic biomarker in human bladder cancer. Mol. Cell. Proteomics.

[12] Dos Santos A, Court M, Thiers V (2010). Identification of cellular targets in human intrahepatic cholangiocarcinoma using laser microdissection and accurate mass and time tag proteomics. Mol. Cell. Proteomics.

[13] Bouamrani A, Ramus C, Gay E (2010). Increased phosphorylation of vimentin in noninfiltrative meningiomas. PLoS One.

[14] Cho WC, Yip TT, Cheng WW (2010). Serum amyloid A is elevated in the serum of lung cancer patients with poor prognosis. Br. J. Cancer.

[15] Vermaat JS, van der Tweel I, Mehra N (2010). Two-protein signature of novel serological markers apolipoprotein-A2 and serum amyloid a predicts prognosis in patients with metastatic renal cell cancer and improves the currently used prognostic survival models. Ann. Oncol..

[16] Xu X, Qiao M, Zhang Y (2010). Quantitative proteomics study of breast cancer cell lines isolated from a single patient: discovery of TIMM17A as a marker for breast cancer. Proteomics.

[17] Xue H, Lü B, Zhang J (2010). Identification of serum biomarkers for colorectal cancer metastasis using a differential secretome approach. J. Proteome Res..

[18] Chang KP, Wu CC, Chen HC (2010). Identif ication of candidate nasopharyngeal carcinoma serum biomarkers by cancer cell secretome and tissue transcriptome analysis: potential usage of cystatin A for predicting nodal stage and poor prognosis. Proteomics.

[19] Tu L, Liu Z, He X (2010). Over-expression of eukaryotic translation initiation factor 4 g 1 correlates with tumor progression and poor prognosis in nasopharyngeal carcinoma. Mol. Cancer.

[20] Feng XP, Yi H, Li MY (2010). Identification of biomarkers for predicting nasopharyngeal carcinoma response to radiotherapy by proteomics. Cancer Res..

[21] Bauer JA, Chakravarthy AB, Rosenbluth JM (2010). Identification of markers of taxane sensitivity using proteomic and genomic analyses of breast tumors from patients receiving neoadjuvant paclitaxel and radiation. Clin. Cancer Res..

[22] Carey MS, Agarwal R, Gilks B (2010). Functional proteomic analysis of advanced serous ovarian cancer using reverse phase protein array: TGF-beta pathway signaling indicates response to primary chemotherapy. Clin. Cancer Res..

[23] Tabernero J, Cer vantes A, Rivera F (2010). Pharmacogenomic and pharmacoproteomic studies of cetuximab in metastatic colorectal cancer: biomarker analysis of a Phase I dose-escalation study. J. Clin. Oncol..

[24] Chung CH, Seeley EH, Roder H (2010). Detection of tumor epidermal growth factor receptor pathway dependence by serum mass spectrometry in cancer patients. Cancer Epidemiol. Biomarkers Prev..

[25] Amann JM, Lee JW, Roder H (2010). Genetic and proteomic features associated with survival after treatment with erlotinib in first-line therapy of non-small cell lung cancer in Eastern Cooperative Oncology Group 3503. J. Thorac. Oncol..

[b26] 26*An Omics Perspective on Cancer Research*. Cho WC (Ed. ). Springer, Berlin, Germany (2010).

[b27] 27Cho WC. Proteinchip. In: *Encyclopedia of Cancer (Volume 3) (2nd Edition)*. Schwab M (Ed. ). Springer, Berlin, Germany, 2475-2478 (2009).

